# How does overweight affect bone mineral density and oral health in adult hypophosphatasia?– A single center experience

**DOI:** 10.1186/s13023-025-03611-9

**Published:** 2025-02-25

**Authors:** Florian Dudde, Dominik Fildebrandt, Karin Petz, Ralf Smeets, Martin Gosau, Michael Amling, Thomas Beikler, Florian Barvencik

**Affiliations:** 1 Department of Osteology and Biomechanics, University Medical Center Hamburg- Eppendorf, Lottestraße 59, 22529 Hamburg, Germany; 2 Department of Periodontics, Preventive and Restorative Dentistry, University Medical Center Hamburg-Eppendorf, Hamburg, Germany; 3 Department of Oral and Maxillofacial Surgery, University Medical Center Hamburg- Eppendorf, Hamburg, Germany; 4 Department of Oral and Maxillofacial Surgery, Division of Regenerative Orofacial Medicine, University Medical Center Hamburg-Eppendorf, Hamburg, Germany

**Keywords:** Adult, Hypophosphatasia, Oral health, BMI

## Abstract

**Aim:**

The aim of this study was to investigate the influence of overweight (BMI ≥ 25 (kg/m²)) on the oral health status in patients with adult hypophosphatasia (HPP).

**Materials and methods:**

Throughout a retrospective assessment both oral health status and bone metabolism including dual x-ray absorptiometry (DXA) for bone mineral density (BMD) measures were analyzed. The oral health status was assessed by the decayed/missing/filled teeth index (DMFT), clinical attachment level (CAL), probing pocket depth (PPD), and the periodontal screening index (PSI). The study population was divided into two groups based on the overweight classification by BMI (Overweight = BMI ≥ 25 kg/m²; *n* = 17) vs. non-overweight ( BMI < 25 kg/m²; *n* = 31).

**Results:**

48 HPP patients were included in this study. Overweight HPP patients showed a significantly reduced oral health status regarding filled teeth, DMFT, PSI, PPD and periodontitis severity index compared to non-overweight HPP patients. Furthermore, overweight HPP patients revealed significantly higher DXA findings regarding BMD, T- and Z-scores.

**Conclusion:**

In the present study overweight (BMI ≥ 25 (kg/m²)) is associated with a poorer oral health status and higher BMD in adult HPP.

**Clinical relevance:**

Since overweight is associated with a poorer oral health status in the general population and promotes the development of periodontal disease, the findings of the present study indicate that overweight also affects oral health in adult HPP.

## Introduction

Hypophosphatasia (HPP) is a rare metabolic disease [1]. This disease is due to a restricted function of the non-tissue-specific alkaline phosphatase (TNSAP), which can be caused by various mutations in the alkaline phosphatase liver/bone/kidney type (ALPL) gene [1, 2]. TNSAP is an important enzyme in bone metabolism and consequently leads to mineralization disorders if its function is impaired [1, 2]. Various studies have shown a wide range of symptoms in HPP [3]. In addition to non-specific symptoms such as arthralgia, myalgia and fatigue, an increased susceptibility to fractures and an increased incidence of atraumatic tooth loss are typical symptoms for HPP [3–5]. Furthermore, initial studies have shown that the oral health status is also disturbed in the sense of increased prevalence/ severity of periodontitis and is associated with increased DMFT (Decayed, Missing, Filled Teeth) indices in HPP [5, 6]. In addition to atraumatic tooth loss, HPP patients often present with enamel hypoplasia, poor cementum formation, and delayed tooth eruption [3–6]. These factors significantly affect oral health by increasing susceptibility to dental caries, periodontal disease, and subsequent tooth loss [5, 6]. Enamel hypoplasia results in a thinner and more brittle enamel structure, predisposing teeth to decay and structural failure [5, 6]. Poor cementum formation compromises the periodontal ligament attachment, leading to an increased risk of atraumatic tooth exfoliation. Delayed eruption of primary and permanent teeth contributes to malocclusion and crowding, further exacerbating the risk of periodontal disease [5, 6]. Collectively, these conditions highlight the multifaceted impact of HPP on oral health.

Although HPP can be classified into various subforms depending on the onset of clinical symptoms almost all HPP patients show a negatively affected oral health status [5–7].

Another risk factor for impaired oral health in the general population is overweight (Body-Mass-Index = BMI ≥ 25 (kg/m²) [8, 9]. Reviews have shown that increased weight promotes the development of periodontitis [10, 11]. Furthermore, a current publication showed that overweight is associated not only with periodontitis but also with the occurrence of decayed teeth and tooth loss [8]. In addition to that, a current meta-analysis reported that individuals with overweight tend to have higher bone mineral density (BMD) values than non-overweight adults [12]. Qiao et al. showed that overweight is positively associated with higher BMD and negatively correlated with the prevalence of osteoporosis [12]. However, if and to what extent overweight affects BMD values in HPP patients has hardly been investigated yet. Since overweight seems to positively correlate with BMD values and negatively with the oral health status in the general population, the question arises whether overweight (BMI ≥ 25 (kg/m²) also affects the oral health status and BMD in adult HPP. Previous work by our group has reported DXA scan results and oral health findings in patients with HPP in the context of laboratory parameters [5]. This study provided important insights into the bone and oral health challenges faced by this population. In the current study, we extend these findings by specifically investigating the influence of overweight on oral health outcomes in adult HPP patients, an area that has not been previously explored.

Due to the rarity of this disease the oral health in adult HPP has rarely been studied.

Furthermore, no study on the correlation of overweight and the oral health status in HPP patients has been published yet. Therefore, the aim of this study was to investigate the influence of overweight on the oral health status and BMD in adult HPP.

## Materials and methods

### Data collection

This retrospective study examined German HPP patients who were treated in the Department of Osteology and Biomechanics at the University Hospital Hamburg and in the Department of Periodontics, Preventive and Restorative Dentistry between 2017 and 2023. Inclusion criteria were a genetically proven adult form of HPP (pathogenic and likely pathogenic variants of ALPL gene, ACMG class III– V (American College of Medical Genetics and Genomics)) and a clear diagnosis of HPP based on the HPP major and minor criteria according to the International Working Group (IWG) on HPP [13, 14]. Data of the respective study population have already been published elsewhere [5]. Exclusion criteria were incomplete medical and/or dental documentation (patient records). Data collection regarding bone metabolism and clinical findings was carried out using the patient files in the Department of Osteology and Biomechanics. The data collection of the oral health status was carried out from the patients’ digital patient files (Charly Program by Solutio, Holzgerlingen, Germany) in the Department of Periodontics, Preventive and Restorative Dentistry. A total of 48 patients were included in this study.

### Clinical data & molecular information

For each patient, the clinical characteristics (age, body-mass-index = BMI, sex, mutation, ACMG class) as well as laboratory parameters (alkaline phosphatase = AP, bone specific alkaline phosphatase = bAP, PLP, Copper, Magnesium, parathyroid hormone = PTH, Vitamin D, Calcium, Phosphate) were analyzed. Furthermore, the results of the dual x-ray absorptiometry (DXA) scan were also analyzed for these patients. Using DXA, measurements of BMD can be determined on the spine, femur (femoral neck) and other specific regions [15]. The values of DXA are presented in bone mineral density, T-Scores and Z-Scores. The intraoral examination was carried out within two weeks after initial diagnosis of HPP and simultaneous serum collection.

### Oral health status

During the routine dental examination the following parameters were investigated:

1. Decayed missing filled/tooth (DMFT) index.
2. Number of natural teeth in situ (excluding third molars).
3. Periodontitis screening index.
4. Periodontitis severity grade (PSI).
5. Probing Pocket Depth (PPD).
6. Clinical attachment level (CAL).

The PPD and CAL were determined using a standardized manual periodontal probe (CP-11; Hu-Friedy, USA). According to Holfreter et al. PPD and CAL were recorded on six sides of the tooth (mesiobuccal, buccal, distobuccal, mesiooral, oral and distooral) [16]. Furthermore the age of the first permanent tooth eruption was noted. The oral assessment was performed by at least two years trained, experienced dentists, in the field of periodontology (DF, KP, TB).

### Overweight classification (BMI)

The present study aimed to analyze the influence of overweight on the oral health status and BMD in adult HPP patients. Consequently, based on the diagnostical criteria for overweight (BMI ≥ 25 (kg/m²)) the study population was divided into two groups (Group A with a BMI ≥ 25 (kg/m²) (*n* = 17) vs. Group B with a BMI < 25 (kg/m²) (*n* = 31)) [8, 9]. A further subgroup analysis by BMI ≥ 25 (kg/m²) (i.e. obesity, extreme obesity) was not performed due to the limited number of HPP patients (*n* = 48).

### Statistical analysis

Descriptive analysis was used to display patients baseline characteristics. Normally distributed continuous variables are presented as mean ± standard deviation and binary variables are using absolute and relative frequencies. Comparison of continuous variables was performed by student’s t-test. Chi-square test was used for analysis of binary variables. Bar plots and scatter plots were used as graphic elements. A p-value < 0.05 was considered statistically significant. All statistical analyses were performed using the SPSS version 28.0 statistical package (IBM, Markham, Canada).

## Results

### Baseline

A total of 48 HPP patients were included in the present study. Based on the diagnostical criteria for overweight (BMI ≥ 25 (kg/m²)) the population was divided into two groups (Group A with a BMI ≥ 25 (kg/m²) (*n* = 17) vs. Group B with a BMI < 25 (kg/m²) (*n* = 31)). Consequently, overweight patients showed significantly higher BMI values than non-overweight patients (Group A = BMI 30.28 (kg/m²) vs. Group B = 21.11 (kg/m²); p = < 0.001) (Table 1). Overweight patients revealed significantly lower Vitamin D levels than non-overweight patients (Group A = 24.27 µg/l vs. Group B = 31.45 µg/l; *p* = 0.047) (Table 1). Consecutively, overweight patients tended to have higher PTH levels with simultaneously reduced calcium and phosphate levels (non-significant) (Table 1). Furthermore, patients in group A showed higher AP and bAP levels with consecutively lower PLP levels (non-significant) (Table 1).

**Table 1:** Patient characteristics (*n* = 48)

Variable	Total (*n* = 48)	BMI ≥ 25 (kg/m²) (*n* = 17)	BMI < 25 (kg/m²) (*n* = 31)	*p*-Value
Age	42.21 (± 15.78)	46.76 (± 14.52)	39.71 (± 16.12)	0.140
Gender				0.161
Male	9 (18.8)	5 (29.4)	4 (12.9)	
Female	39 (81.3)	12 (70.6)	27 (87.1)	
BMI (kg/m²)	24.35 (± 5.86)	30.28 (± 5.52)	21.11 (± 2.66)	**< 0.001**
ACMG Class				0.112
1	0 (0)	0 (0)	0 (0)	
2	0 (0)	0 (0)	0(0)	
3	16 (33.3)	2 (11.8)	14 (45.2)	
4	8 (16.7)	3 (17.6)	5 (16.1)	
5	24 (50.0)	12 (70.6)	12 (38.7)	
PLP (µg/l)	87.10 (± 68.31)	68.15 (± 44.04)	96.75 (± 98.38)	0.179
AP (U/l)	33.83 (± 20.83)	36.52 (± 24.95)	28.94 (± 8.21)	0.232
bAP (U/l)	5.93 (± 7.23)	6.68 (± 8.82)	4.56 (± 2.21)	0.336
Magnesium (mmol/l)	0.85 (± 0.07)	0.88 (± 0.06)	0.84 (± 0.07)	0.090
Copper (µg/l)	1075.55 (± 395.43)	1175.13 (± 340.51)	1010.61 (± 421.93)	0.214
Calcium (mmol/l)	2.37 (± 0.12)	2.35 (± 0.13)	2.41 (± 0.11)	0.131
Phosphate (mmol/l)	1.14 (± 0.26)	1.12 (± 0.28)	1.19 (± 0.21)	0.415
Vitamin D (µg/l)	28.98 (± 15.39)	24.47 (± 17.49)	31.45 (± 13.78)	**0.047**
PTH (ng/l)	44.15 (± 18.82)	49.39 (± 21.34)	41.27 (± 17.04)	0.155

Note: Data are presented as mean ± SD and/or percentage

Abbreviations: AP = alkaline phosphatase, bAP = bone specific alkaline phosphatase, BMI = Body-mass-index, ACMG = American College of Medical Genetics, PLP = Pyridoxal phosphate, PTH = Parathyroid hormone

### DXA-Scan

There were significant differences between the two groups regarding the DXA findings. Overweight HPP patients showed significantly higher T- and Z-scores for both femora and the spinal column (Table 2). BMD was significantly increased, particularly for both femora in overweight HPP patients (Table 2).

**Table 2:** DXA scan (SD)

Variable	Total (*n* = 48)	BMI ≥ 25 (kg/m²) (*n* = 17)	BMI < 25 (kg/m²) (*n* = 31)	*p*-Value
BMD SC (g/cm²)	1.14 (± 0.22)	1.27 (± 0.19)	1.08 (± 0.17)	0.232
T-Score SC	− 0.16 (± 1.59)	0,72 (± 1.31)	− 0.75 (± 1.34)	**0.003**
Z-Score SC	− 0.03 (± 1.33)	0.63 (± 1.31)	− 0.42 (± 1.20)	**0.008**
BMD Left Femur (g/cm²)	0.95 (± 0.15)	1.05 (± 0.15)	0.89 (± 0.12)	**< 0.001**
T-Score Left Femur	− 0.49 (± 1.10)	0.16 (± 1.08)	− 0.92 (± 0.91)	**0.002**
Z-Score Left Femur	− 0.21 (± 0.98)	0.26 (± 1.08)	− 0.49 (± 0.82)	**0.011**
BMD Right Femur (g/cm²)	0.94 (± 0.14)	1.03 (± 0.14)	0.89 (± 0.11)	**< 0.001**
T-Score Right Femur	− 0.57 (± 1.00)	0.01 (± 0.89)	− 0.95 (± 0.90)	**0.002**
Z-Score Right Femur	− 0.28 (± 0.95)	0.14 (± 1.00)	− 0.53 (± 0.84)	**0.022**

Note: Data are presented as mean ± SD

SC = Spinal column, BMD = Bone mineral density

### Oral health status– DMFT

**Fig. 1:**
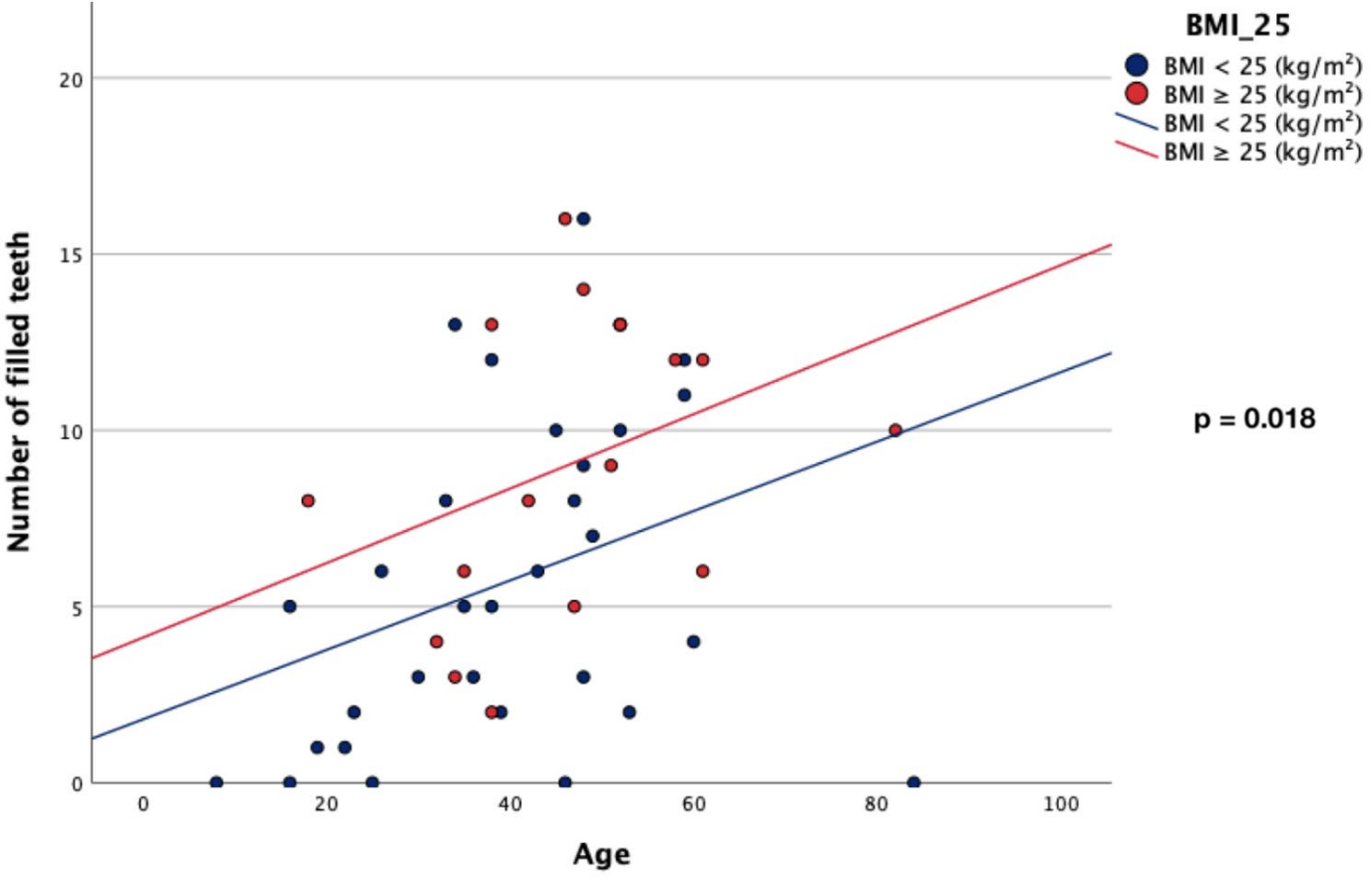
Scatter plot– number of filled teeth– correlation of age and BMI (kg/m²)

Overweight HPP patients revealed a lower number of natural teeth than non-overweight HPP patients (Table 3). Overweight HPP patients showed higher numbers of decayed and missing teeth than non-overweight HPP patients (non-significant) (Table 3). Significant differences were found for the number of filled teeth between the two groups (Group A = 9.06 vs. Group B = 5.71; *p* = 0.018) (Table 3; Fig. 1). Consecutively, overweight HPP patients showed a significantly higher DMFT-Index than non-overweight HPP patients (Group A = 14.65 vs. Group B = 10.29; *p* = 0.025) (Table 3).

**Table 3:** Oral health status– DMFT in HPP patients (SD)

Variable	Total (*n* = 48)	BMI ≥ 25 (kg/m²) (*n* = 17)	BMI < 25 (kg/m²) (*n* = 31)	*p*-Value
Number of natural permanent teeth	25.21 (± 3.94)	24.82 (± 3.64)	25.42 (± 4.13)	0.621
Decayed	2.15 (± 2.44)	2.41 (± 2.85)	2.00 (± 2.22)	0.291
Missing	2.79 (± 3.94)	3.18 (± 3.64)	2.58 (± 4.13)	0.331
Filled	6.90 (± 4.76)	9.06 (± 4.24)	5.71 (± 4.67)	**0.018**
DMFT-Index	11.83 (± 6.53)	14.65 (± 5.78)	10.29 (± 6.49)	**0.025**

Note: Data are presented as mean ± SD

DMFT = Decayed-Missing-Filled-Teeth-Index

### Oral health status– periodontal status

**Fig. 2:**
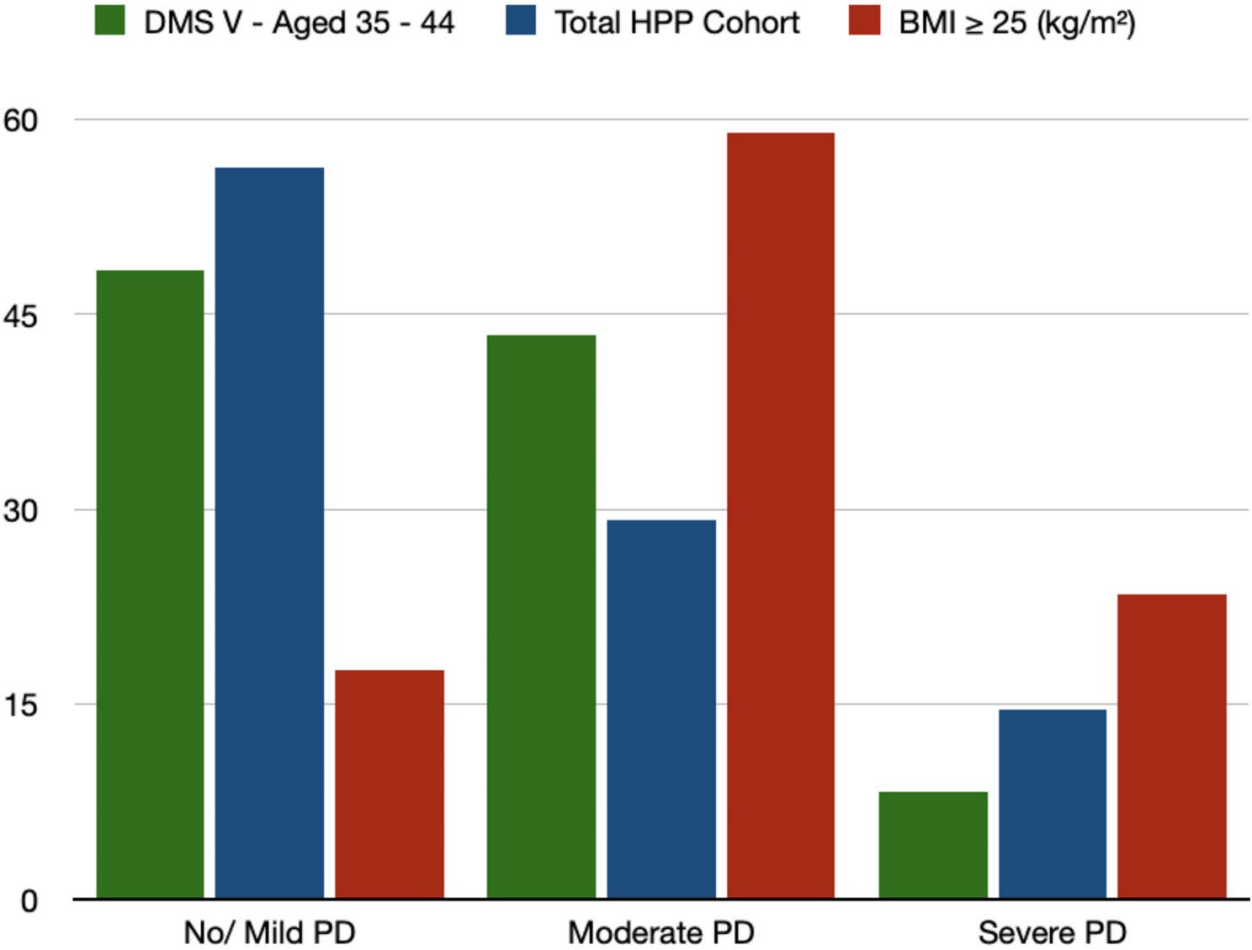
Bar plot– comparison of periodontitis severity grade with DMS-V findings [17, 18].

Note: Data are presented as percentage. Abbreviations: PD = Periodontitis, BMI = Body-Mass-Index, DMS-V = Deutsche Mundgesundheitsstudie V, HPP = Hypophosphatasia.

There were significant differences between the two groups regarding the periodontal status (Table 4). Overweight HPP patients showed significantly higher PSI values in all sextants in comparison with non-overweight HPP patients (Table 4). Overweight HPP patients also showed higher relative frequencies of sites and teeth per mouth with a CAL > 3 mm and > 5 mm than non-overweight HPP patients (non-significant) (Table 4). There were significant differences between the two groups, particularly regarding PPD (Table 4). HPP patients with a BMI ≥ 25 (kg/m²) showed significantly more sites and teeth per mouth with a PPD > 4 mm / 6 mm than HPP patients with a BMI < 25 (kg/m²) (Table 4). Consequently, overweight HPP patients showed significantly worse periodontitis severity scores than non-overweight HPP patients (Table 4; Fig. 2).

**Table 4:** Detailed periodontal data (SD and/or percentage) in HPP patients

Variable	Total (*n* = 48)	BMI ≥ 25 (kg/m²) (*n* = 17)	BMI < 25 (kg/m²) (*n* = 31)	*p*-Value
PSI - S1	2.19 (± 1.02)	2.71 (± 0.85)	1.90 (± 1.02)	**0.008**
PSI - S2	1.94 (± 0.93)	2.41 (± 0.80)	1.68 (± 0.91)	**0.008**
PSI - S3	2.13 (± 1.04)	2.76 (± 0.83)	1.77 (± 0.99)	**0.001**
PSI - S4	2.04 (± 0.97)	2.53 (± 0.87)	1.77 (± 0.92)	**0.007**
PSI - S5	2.10 (± 1.03)	2.47 (± 0.72)	1.90 (± 0.79)	**0.018**
PSI - S6	1.92 (± 1.03)	2.35 (± 1.00)	1.68 (± 0.98)	**0.028**
Sites per Mouth CAL > 3 mm (%)	11.80 (± 12.62)	14.95 (± 13.16)	10.08 (± 12.18)	0.204
Sites per Mouth CAL > 5 mm (%)	2.31 (± 6.10)	2.61 (± 5.99)	2.15 (± 6.26)	0.806
Teeth per Mouth CAL > 3 mm (%)	13.10 (± 12.09)	16.35 (± 12.67)	11.31 (± 11.58)	0.169
Teeth per Mouth CAL > 5 mm (%)	2.38 (± 5.41)	2.79 (± 5.58)	2.15 (± 5.40)	0.703
Sites per Mouth PPD > 4 mm (%)	11.55 (± 19.11)	19.44 (± 22.63)	7.22 (± 15.63)	**0.033**
Sites per Mouth PPD > 6 mm (%)	3.69 (± 9.70)	6.81 (± 12.44)	1.98 (± 7.50)	**0.009**
Teeth per Mouth PPD > 4 mm (%)	20.30 (± 24.09)	32.81 (± 28.65)	13.38 (± 18.22)	**0.006**
Teeth per Mouth PPD > 6 mm (%)	4.29 (± 11.36)	8.52 (± 16.51)	1.97 (± 6.40)	**0.005**
Age at first tooth exfoliation	5.06 (± 0.63)	5.00 (± 0.63)	5.18 (± 0.63)	0.361
Periodontitis Severity Grade*				**< 0.001**
No/mild PD	27 (56.3)	3 (17.6)	24 (77.4)	
Moderate PD	14 (29.2)	10 (58.9)	4 (12.9)	
Severe PD	7 (14.6)	4 (23.5)	3 (9.7)	

Note: Data are presented as mean ± SD and/or percentage

Abbreviations: PSI = Periodontitis screening index per sextant (S2– S6), CAL = Clinical attachment level, PPD = Probing pocket depth, PD = Periodontitis, * Eke PI, et al. [17]

## Discussion

HPP is a rare metabolic disease with varying symptoms [1, 2]. Recently, it was reported that most HPP patients also show dental manifestations of this entity [4–6]. However, the study situation on the oral health status in HPP is rare [5, 6]. In the present study, the influence of overweight on oral health status and BMD was examined in patients with genetically confirmed adult HPP.

Overweight is a well-studied risk factor for a variety of diseases [19]. Current studies have shown that overweight is associated not only with heart and circulatory diseases, but also with poorer oral health status [8, 10, 11]. Current publications showed that overweight can be associated in particular with increased DMFT indices, increased numbers of decayed teeth and an increased prevalence of periodontitis [8, 10]. Particularly regarding the DMFT index and the number of decayed teeth, the results of the present study are in line with the current literature, revealing that overweight is also associated with an increased prevalence of decayed and filled teeth in HPP patients [20]. In the present study, overweight HPP patients showed significantly higher numbers of filled teeth and a consecutively increased DMFT index compared to non-overweight HPP patients. Possible pathophysiological reasons that lead to decayed and filled teeth in the context of overweight include excessive consumption of foods containing high amounts of sugar, as overweight can often be due to unhealthy and/or disordered eating behavior [21, 22].

In particular, the frequent and excessive consumption of food containing a lot of sugar (low molecular carbohydrates), and the presence of cariogenic microorganisms (i.e. *Streptococcus mutans*) coupled with inadequate tooth brushing technique and insufficient topical fluoride application can lead to a disruption in the balance of demineralization and remineralization of the dental structures (i.e. enamel) [23–25]. Regular and correct dental care can prevent the onset and progression of tooth decay. Furthermore, emerging evidence highlights the association between overweight and changes in the oral microbiome, often referred to as dysbiosis [26]. Overweight and obesity are linked to systemic low-grade inflammation, which may alter the composition of the oral microbiome [26]. Dysbiosis can disrupt the balance between commensal and pathogenic bacteria, increasing susceptibility to oral diseases such as periodontitis and caries [25]. This phenomenon is supported by recent findings indicating that metabolic disorders can exacerbate inflammatory responses within the oral cavity, potentially through mechanisms involving microbial imbalance [26]. In the context of HPP, where patients are already predisposed to oral health issues due to defective mineralization, dysbiosis could further amplify the oral disease burden. While our study did not directly assess the oral microbiota, its potential role as a mediator between overweight and oral health outcomes represents an important avenue for future research.

Focusing on high BMI-levels, Baskaradoss et al. was able to show that adolescents with a metabolic syndrome tended to brush their teeth less frequently compared to adolescents without a metabolic syndrome in a longitudinal study [27]. In addition to that, caries is strongly influenced by sugar intake from energy-rich foods, which also play a crucial role in the development of overweight [28]. Furthermore, it is known that caries and overweight are often associated with a lower socioeconomic status [29].

In addition, overweight leads to a dysbalance between anti- and pro-inflammatory cytokines [8]. Issrani et al. revealed that, particularly in the context of overweight, increased concentrations and sometimes uncontrolled release of proinflammatory cytokines (i.e. IL-1β (Interleukin), TNF-a (Tumor-necrosis factor), Prostaglandin E2) can be observed [8, 30, 31]. These proinflammatory cytokines are crucial in the development and maintenance of periodontitis [8]. Consequently, studies have shown that overweight patients revealed higher prevalences and increased severity of periodontitis than non-overweight individuals in the general population [8, 30, 31]. In a recent publication Cetin et al. was able to detect a direct correlation between BMI and clinical attachment loss, probing pocket depth, plaque index, stage and grade of periodontitis, and the number of remaining teeth [8, 32]. In their study it was stated that BMI increases the risk of developing severe stages of periodontitis [8, 32].

These findings were also demonstrated in the present study. Overweight HPP patients showed significantly higher numbers of sites and teeth per mouth with PPD > 4 mm and > 6 mm than non-overweight HPP patients. Furthermore, severe grades of periodontitis were significantly more common in overweight HPP patients in direct comparison with non-overweight HPP patients. Even in comparison with the results of the German Oral Health Study-V (DMS-V, age-correlated healthy normal population aged 35–44), the rates of severe periodontitis of all HPP patients but especially those of overweight HPP patients exceeded the results of the age-matched general population clearly (Fig. 2) [17, 18]. Current studies have been able to demonstrate that the oral health status in HPP correlates with various biochemical markers [5, 6]. Weider et al. demonstrated that the number of ALPL mutations correlates significantly with a negative oral health status [6].

Furthermore, it was proven that the oral health status is consecutively associated with reduced TNSAP activity and subsequently increased substrate levels of pyridoxal-5-phosphate [5]. However, since in the present study overweight HPP patients tended to have lower levels of PLP than non-overweight HPP patients, BMI seems to have a more lasting influence on oral health status than initially assumed.

The relationship between BMI and oral health-related quality of life (OHRQoL) is increasingly recognized in the literature. Overweight and obesity have been associated with negative perceptions of oral health, potentially due to higher susceptibility to periodontal disease and caries, as well as psychosocial factors such as self-esteem and social interactions [33]. In the context of HPP, the additional burden of oral diseases, exacerbated by overweight status, may further diminish OHRQoL [33]. While our study primarily assessed clinical oral health outcomes, the impact of these outcomes on patients’ perceived quality of life is a critical consideration for future research. Integrating patient-reported outcomes, such as OHRQoL scores, could provide a more comprehensive understanding of the interplay between BMI, oral health, and overall well-being in this population.

Another laboratory factor influencing oral health is the Vitamin D level. Since Vitamin D deficiency is also associated with poorer oral health in the general population and further promotes the occurrence of periodontitis, the influence of this key hormone should not be left unmentioned regarding HPP [17, 34, 35]. In the present study, overweight HPP patients revealed significantly lower levels of Vitamin D than non-overweight HPP patients (data not shown). Consequently, a lower Vitamin D level needs to be considered at least a cofactor for the poor periodontal status (i.e. PPD, PSI) and appears to aggravate the worse oral health status in HPP.

In the present study, overweight HPP patients had significantly higher T-scores and Z-scores in the spinal column and both femora. Significantly higher BMD was recorded in both femora. A current meta-analysis reported that individuals with overweight tend to have higher BMD values than non-overweight individuals [12]. Qiao et al. showed that overweight was positively associated with higher BMD and negatively correlated with the prevalence of osteoporosis in comparison with non-overweight individuals [12].

In HPP, the complete effects of impaired bone metabolism on BMD are not fully understood. Genest et al. showed that BMD in adult HPP patients is not systematically reduced [36]. Hepp et al. examined biochemical and clinical parameters of HPP patients in their cross-sectional study and compared them to healthy non-HPP patients [37]. Here, BMD as well as parameters of body composition did not differ between adults with HPP and the control group [37]. Since there is a lack of studies investigating the influence of overweight on BMD in HPP patients, the current findings revealed a similar association between BMI and BMD as in the general population. Therefore, overweight seems to increase BMD in HPP despite the fact that the overweight group showed lower Vitamin D levels which is generally associated with impaired bone metabolism and a higher risk of osteoporosis [38]. The observation that overweight appears to increase BMD in HPP despite lower Vitamin D levels may be explained by several mechanisms in our opinion. Increased mechanical loading in overweight individuals can stimulate bone formation, potentially leading to higher BMD. Additionally, adipose tissue-derived hormones such as leptin might positively influence bone metabolism. While Vitamin D deficiency is generally associated with impaired bone metabolism and increased osteoporosis risk, the metabolic adaptations seen in overweight individuals, such as greater nutrient availability or compensatory mechanisms, may counterbalance these effects. These findings underscore the complexity of bone metabolism in HPP and highlight the need for further research into the interplay between metabolic status, Vitamin D, and bone health in this population. The current findings are limited to several aspects. The present study was conducted as a monocentric study with a limited population size. In addition to that, this study is retrospective in nature and therefore without follow-up protocol. Hence, the long-term influence of HPP and overweight adjustment on the oral health status and BMD was not examined. Nevertheless, it can be concluded that overweight (BMI ≥ 25 (kg/m²) correlates with a poorer oral health status and higher BMD when compared to non-overweight HPP patients. Significant differences were found, regarding decayed teeth, DMFT-Index, PPD, periodontitis severity index, T-Scores and Z-Scores in all regions and BMD values for both femora. The present study represents the first publication that revealed a negative influence of overweight on the oral health status in HPP patients.

While these findings highlight the potential impact of metabolic factors on oral health, the retrospective design of the study precludes causal inferences. Confounding factors, such as dietary habits, systemic inflammation, and socioeconomic influences, may contribute to the observed associations.

Nevertheless, the findings of this study highlight the importance of considering metabolic factors, such as overweight, in the management of oral health in patients with HPP. Overweight HPP patients exhibited significantly poorer periodontal health and increased caries prevalence, suggesting that addressing weight management could be a valuable strategy for reducing oral disease burden. Clinicians should adopt a multidisciplinary approach, integrating dental care with metabolic and nutritional counseling to optimize outcomes in this vulnerable population. Furthermore, targeted periodontal therapies and preventive measures, such as tailored oral hygiene education and regular dental check-ups, are critical for mitigating the impact of these oral health challenges.

This study underscores the need for further research to elucidate the mechanisms linking overweight to oral health in HPP patients. Key areas for future investigation include the role of the oral microbiota in mediating these associations, the impact of systemic inflammation on periodontal disease progression, and the interplay between metabolic and genetic factors in HPP. Incorporating longitudinal study designs and advanced tools, such as microbiome analysis and patient-reported outcomes like OHRQoL, could provide deeper insights into these relationships and inform personalized treatment approaches. In addition to the recent findings, further studies with larger HPP populations are needed to confirm these results and to examine the influence of overweight as well as (extreme) obesity on the oral health status in HPP patients in more detail.

## Data Availability

The data that support the findings of this study are not openly available due to reasons of sensitivity and are available from the corresponding author upon reasonable request.
